# Supplementary material for: DNA Adenine Methylation Clock in Brain Aging and Alzheimer’s Disease Progression
Source: bioRxiv. 2026 Mar 6:2026.03.05.709867. Preprint. [Version 1] doi: 10.64898/2026.03.05.709867 (PMC13001325; doi:10.64898/2026.03.05.709867)
Supplement: Supplement 1 [file media-1.pdf]

Supplementary Information file for

DNA Adenine Methylation Clocks in Brain Aging and Alzheimer's Disease Progression

Abdur Rahim[1], [¥] Xiaomei Zhan[5], [¥] Qiyuan Han[2], Allysha O'Donnell[3], Angela Jeong[4], Guru-Swamy Madugundu[1], Suresh Pujari[1], Monica Kruk[1], Xinlong Luo[5], Ling Li[4], Tao P. Wu[5,*] Natalia Y. Tretyakova[1*]

[1] Department of Medicinal Chemistry, College of Pharmacy, University of Minnesota-Twin Cities, Minneapolis, MN, 55455, USA

[2] Department of Biochemistry, Molecular Biology and Biophysics, University of Minnesota-Twin Cities, Minneapolis, MN, 55455, USA

[3] Department of Chemistry, University of Minnesota-Twin Cities, Minneapolis, MN, 55455, USA

[4] Department of Experimental and Clinical Pharmacology, University of Minnesota-Twin Cities, Minneapolis, MN, 55455, USA

[5] Department of Molecular and Human Genetics, Baylor College of Medicine, Houston, TX, 77030, USA

*Correspondence: trety001@umn.edu and Tao.Wu@bcm.edu

[¥]These two authors contributed equally

Figure S1: NAME-seq results for N^{6}medA in genomic DNA isolated from prefrontal cortex of 3 human subjects: a young female (19 y.o.), a senior female control (81.8 y.o), a female with mild cognitive impairment (MCI, 82.9 y.o.), and a female AD patient (82.5 y.o). **a-d,** NAME-seq results for GO enrichment analysis on genes containing N^{6}medA peaks in AD female, senior healthy female and young healthy female respectively. **e-f,** Top sequence motif for adenine methylation in mild cognitive impairment patient (MCI) and senior healthy respectively.

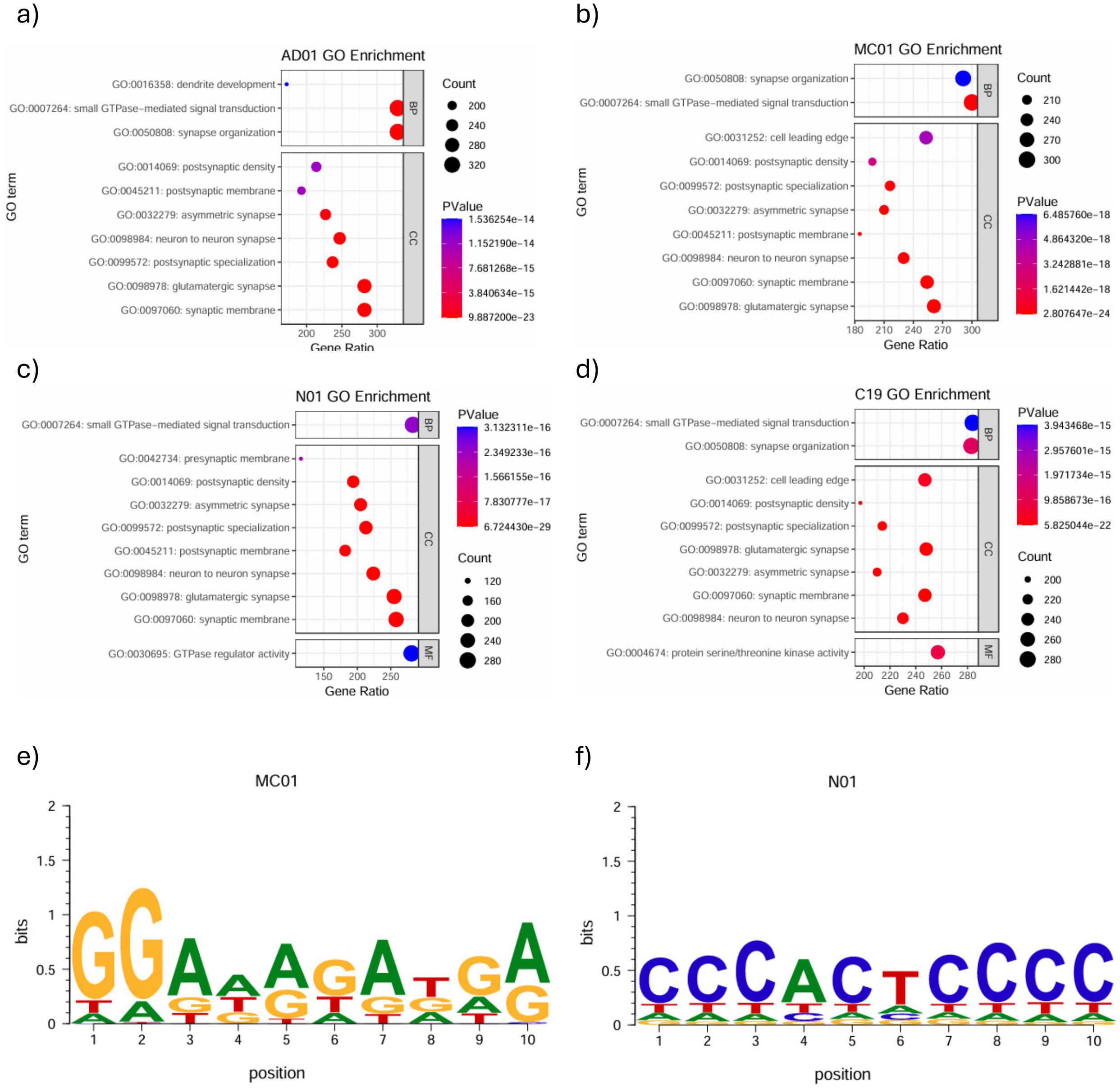

Figure S2a-c: Cross validation of N^{6}medA peaks using MeDIP-seq and NAME seq methodologies in genomic DNA isolated from prefrontal cortex of 3 human subjects: a female AD patient (82.5 y.o), a senior female control (81.8 y.o), and a young female (19 y.o.). Approximately 10% of N^{6}medA sites were identified in NAME seq were cross valided by MeDIP seq.

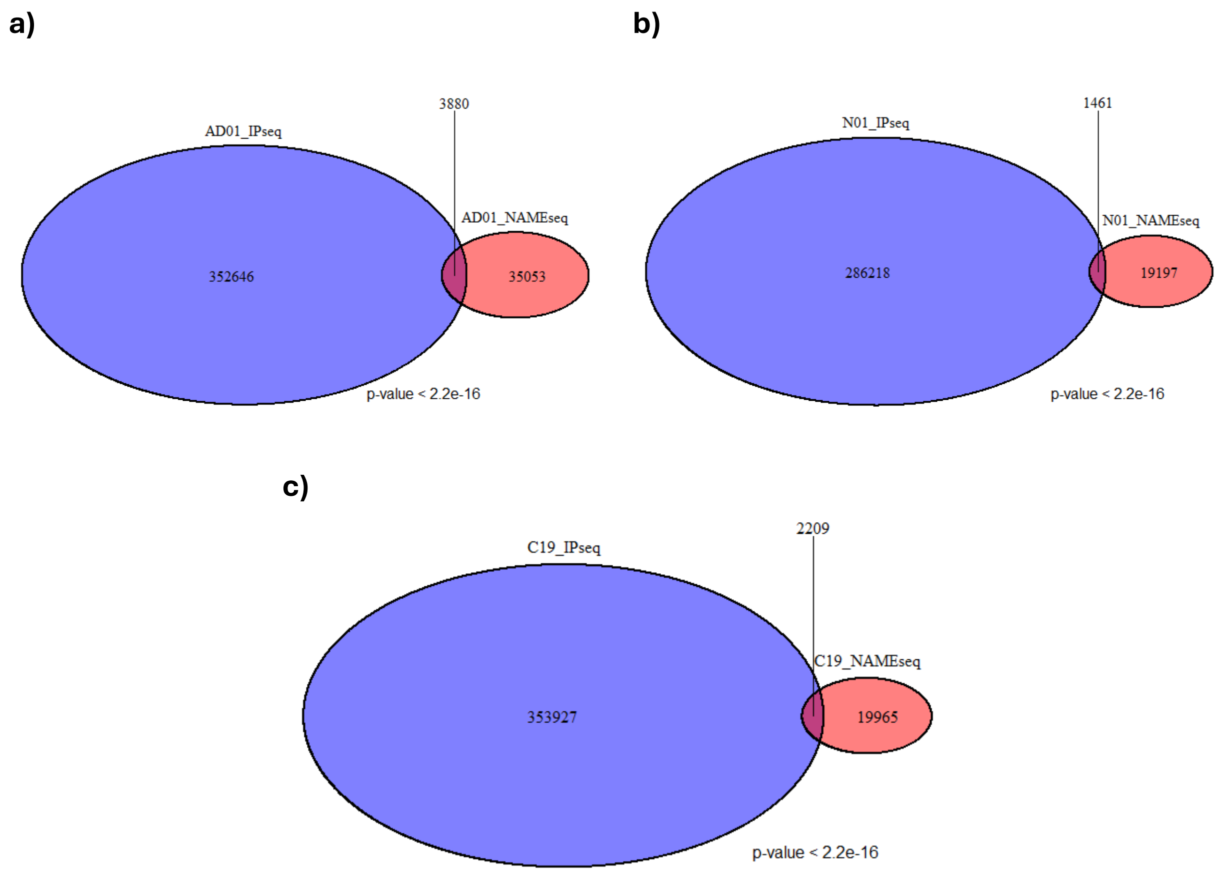

Figure S3: Integration of MeDIP-seq and NAME-seq results for N⁶medA in genomic DNA isolated from prefrontal cortex of 3 human subjects: a young female (19 y.o.), a senior female control (81.8 y.o), a female with mild cognitive impairment (MCI, 82.9 y.o.), and a female AD patient (82.5 y.o).

a-d, integration results for GO enrichment analysis on genes containing N⁶medA peaks in AD female, senior healthy female and young healthy female respectively. **e-f,** integration results for top sequence motif for adenine methylation in senior healthy female and young healthy female respectively.

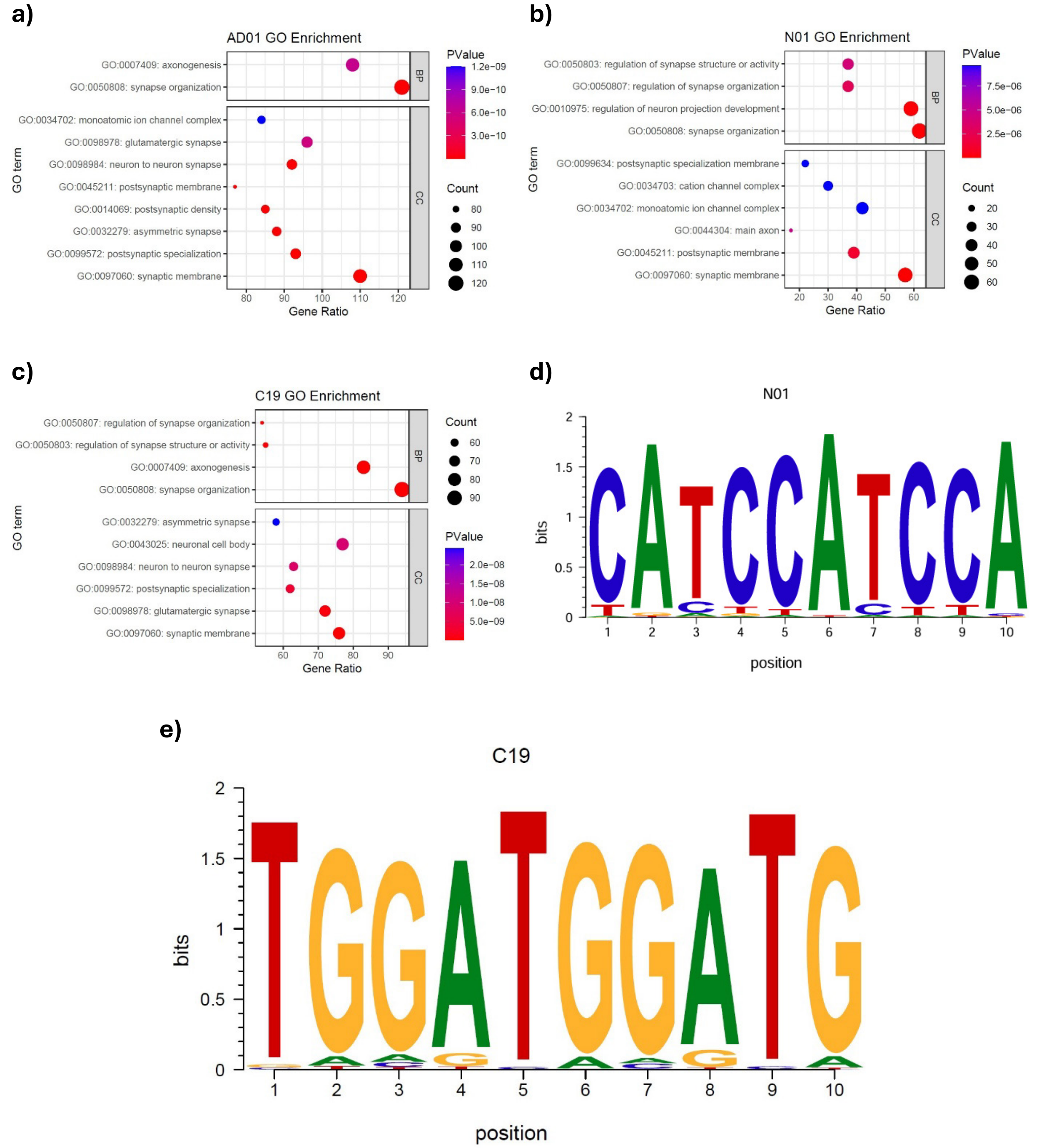

Figure S4: Characterization of protein readers of N⁶medA via affinity proteomics for DNA repair pathway. a, volcano plot showing the quantitative analysis of proteins identified in DNA repair pathway. **b,** Hierarchical clustering heatmap showing top 25 protein abundance identified in DNA repair pathway upon N⁶medA affinity pulldown in SH-SY5Y cells nuclear extract.

a)

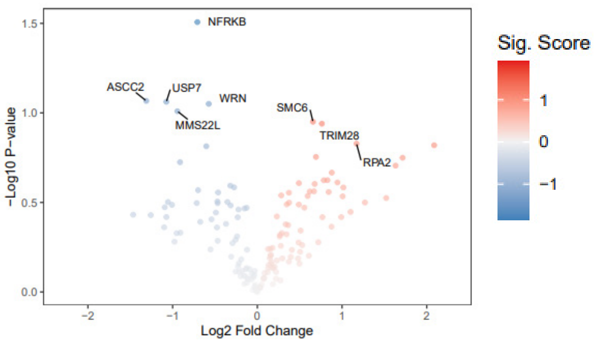

b)

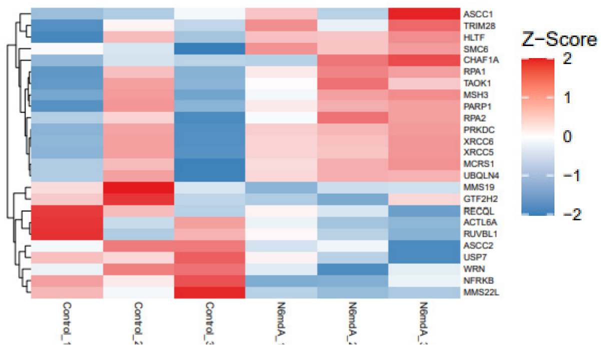

Table S1: Number of N⁶medA sites per cell calculated using different methods

Method	N⁶medA sites per cell
LC-MS	~5700
NAME-seq	~30000
MeDIP-seq	~300000
Overlap	~3000
